# Hybrid cardiac telerehabilitation for coronary artery disease in Australia: a cost-effectiveness analysis

**DOI:** 10.1186/s12913-023-09546-w

**Published:** 2023-05-20

**Authors:** Sameera Senanayake, Ureni Halahakone, Bridget Abell, Sanjeewa Kularatna, Victoria McCreanor, Steven M. McPhail, Julie Redfern, Tom Briffa, William Parsonage

**Affiliations:** 1 Australian Centre for Health Services Innovation and Centre for Healthcare Transformation, School of Public Health and Social Work, Faculty of Health, Queensland University of Technology (QUT), 60 Musk Ave, Kelvin Grove, QLD 4059 Australia; 2 Digital Health and Informatics, Metro South Health, Brisbane, QLD Australia; 3 School of Health Sciences, Faculty of Medicine and Health and Charles Perkins Centre, The University of Sydney, Sydney, Australia; 4 The George Institute for Global Health, University of New South Wales, Sydney, Australia; 5 School of Population and Global Health, University of Western Australia, Perth, Australia; 6 Royal Brisbane and Women’s Hospital, Metro North Health, Herston, QLD Australia

**Keywords:** Cardiac rehabilitation, Hybrid, Cost-effectiveness, Telerehabilitation

## Abstract

**Background:**

Traditional cardiac rehabilitation programs are centre-based and clinically supervised, with their safety and effectiveness well established. Notwithstanding the established benefits, cardiac rehabilitation remains underutilised. A possible alternative would be a hybrid approach where both centre-based and tele-based methods are combined to deliver cardiac rehabilitation to eligible patients. The objective of this study was to determine the long-term cost-effectiveness of a hybrid cardiac telerehabilitation and if it should be recommended to be implemented in the Australian context.

**Methods:**

Following a comprehensive literature search, we chose the Telerehab III trial intervention that investigated the effectiveness of a long-term hybrid cardiac telerehabilitation program. We developed a decision analytic model to estimate the cost-effectiveness of the Telerehab III trial using a Markov process. The model included stable cardiac disease and hospitalisation health states and simulations were run using one-month cycles over a five-year time horizon. The threshold for cost-effectiveness was set at $AU 28,000 per quality-adjusted life-year (QALY). For the base analysis, we assumed that 80% completed the programme. We tested the robustness of the results using probabilistic sensitivity and scenario analyses.

**Results:**

Telerehab III intervention was more effective but more costly and was not cost-effective, at a threshold of $28,000 per QALY. For every 1,000 patients who undergo cardiac rehabilitation, employing the telerehabilitation intervention would cost $650,000 more, and 5.7 QALYs would be gained, over five years, compared to current practice. Under probabilistic sensitivity analysis, the intervention was cost-effective in only 18% of simulations. Similarly, if the intervention compliance was increased to 90%, it was still unlikely to be cost-effective.

**Conclusion:**

Hybrid cardiac telerehabilitation is highly unlikely to be cost-effective compared to the current practice in Australia. Exploration of alternative models of delivering cardiac telerehabilitation is still required. The results presented in this study are useful for policymakers wanting to make informed decisions about investment in hybrid cardiac telerehabilitation programs.

**Supplementary Information:**

The online version contains supplementary material available at 10.1186/s12913-023-09546-w.

## Introduction

Cardiac rehabilitation is a holistic multidisciplinary program tailored to provide patients with cardiovascular disease a range of supports including education, risk factor management, psychosocial care, eating healthy and exercise training [1]. Conventional cardiac rehabilitation programs are centre-based under clinical supervision, with their safety and effectiveness well established. For example, cardiac rehabilitation significantly improves quality of life, reduces cardiac hospitalisations and mortality, and in turn reduces costs to the healthcare system [2]. Given these benefits, referral to cardiac rehabilitation is a Class 1A recommendation for secondary prevention of cardiovascular disease [3]. However, current programs remain underutilised world-wide [4] with reported attendance rates as low as 18–40% [5]. Two Australian studies reported that only 30% of patients eligible for cardiac rehabilitation were referred and fewer than one-third of those referred attended the program [6, 7].

Low referral and participation rates can be attributed to several issues that occur across patient, provider, program and system levels. These include gender, socio-economic factors [8], travel time and distance to centre-based cardiac rehabilitation facilities [9], poor referral systems [10], fragmented care [11], and out-of-pocket costs of attending programs [12]. Low referral and attendance rates indicate that the benefits cardiac rehabilitation provides in the secondary prevention of cardiovascular disease are underutilised in Australia. As such, alternative models of care are required to supplement centre-based programs to encourage increased participation, adherence and completion.

A number of different cardiac rehabilitation models have been trialled to improve uptake. In particular, telerehabilitation, or the delivery of services via telecommunication networks or the internet, has garnered much attention for its potential to overcome some of the barriers of conventional centre-based cardiac rehabilitation. Additionally, telerehabilitation has demonstrated effectiveness [13], may reduce healthcare costs associated with rehospitalisation and absenteeism from work [14], and can be delivered in convenient ways for patients. Despite the increasing availability of such approaches, few were implemented before the pandemic. However, the rapid transition of services to virtual cardiac rehabilitation across Australia in 2020 highlighted the feasibility and acceptability of remote program delivery [15]. Implementation of fully tele-based cardiac rehabilitation programs however, comes with its own set of challenges including increased staff workload, as well as requirements for digital literacy, access to equipment, system infrastructure, and technological support [16]. A possible alternative would be a hybrid approach where both centre-based and tele-based methods are combined to deliver cardiac rehabilitation to eligible patients. When the willingness to participate in a cardiac telerehabilitation program was assessed amongst cardiac rehabilitation participants, the majority (70%) indicated that they would be interested in a hybrid cardiac rehabilitation program [17]. Additionally, delivering telehealth alongside centre-based programs has been reported to be the preference of Australian cardiac rehabilitation providers [16]. Moreover, adding independent home-based rehabilitation to supervised centre-based care may encourage patients to improve self-management of their condition and promote long-term behaviour change. However, trials of such hybrid programs have reported mixed results, and none have evaluated if hybrid cardiac telerehabilitation programs sustain benefits beyond the study period [18, 19].

In the absence of randomised controlled trials with sustained follow-up, there has been increasing use of decision analytic models to determine the long-term costs and benefits of a health intervention. Decision analytic models integrate information from various sources into a single analytical framework and capture the variation of economic outcomes over a long period of time. Consequently, model-based economic evaluations provide better evidence for decision making than trial-based economic evaluations. Even though model-based economic evaluations are vital in identifying cost-effective interventions, none of the systematic reviews to date have assessed the long-term cost-effectiveness of a hybrid cardiac telerehabilitation program. Therefore, the objective of this study was to determine the long-term cost-effectiveness of a hybrid cardiac telerehabilitation in the context of implementation in the Australian setting.

## Methods

### Intervention selection

As cost-effectiveness studies require detailed costing information such as the intervention cost, cost of the health service utilisation, and long-term effectiveness measures, the first aim was to find a study that met the above requirements. To do this, previously published systematic reviews examining the cost-effectiveness of cardiac rehabilitation were used [20, 21] to identify a trial which had used a hybrid cardiac telerehabilitation program as the intervention. An electronic database search was conducted for the time period 2017 to January 2022 using the same terms as in Scherrenberg et al. [20], to identify new studies relevant to these reviews.

This process identified several potential randomised controlled trials which measured costs of telerehabilitation for cardiovascular disease (Supplementary table 1). Three of these studies [22–24] reported on cardiac telerehabilitation in the Australian/New Zealand context. All aimed to determine the effectiveness of home-based remotely monitored telerehabilitation programs compared to conventional centre-based care. While these studies could have provided contextually relevant interventions for our analytical model, none reported on the probability of cardiac related hospitalisation after cardiac rehabilitation which is an important health service outcome in determining the cost-effectiveness of an intervention. Most other international trials identified also failed to report enough information for developing a decision analytic model. For example, they lacked information that could be used to determine health state transition probabilities or information on health service utilisation. Other potential studies were ruled out as they focussed mostly on patients with heart failure or were conducted too long ago to be applicable to current practice.

Consequently, based on these results and expert opinion of clinical members of the team, the Belgium Telerehab III [25] was the only suitable intervention to model in our analysis. Telerehab III was a multicentred randomised control trial, that investigated the effectiveness of a long-term hybrid cardiac telerehabilitation program. The trial based economic evaluation of the Telerehab III study reported that hybrid cardiac telerehabilitation was more effective in reducing cardiac related hospitalisations compared to conventional centre-based cardiac rehabilitation alone. Furthermore, health benefits persisted when the Telerehab III outcomes were reported after 2 years follow up [26].

Therefore, a Markov model was developed to assess the cost-effectiveness of Australians who undergo hybrid cardiac telerehabilitation (Telerehab III trial) compared to those attending centre-based cardiac rehabilitation.

### Telerehab III trial

The Telerehab III trial comprised of 140 patients, who had (i) coronary heart disease (94% in the intervention group and 93% in the control group), treated with coronary artery bypass grafting or percutaneous coronary intervention or (ii) chronic heart failure (6% in the intervention group and 7% in the control group) with reduced or preserved ejection fraction and defined by the New York Heart Association (NYHA) functional class system I to III only [25].

The intervention group (*n* = 70) were enrolled in a 24-week cardiac telerehabilitation program after receiving the first 6 weeks of a standard 12-week centre-based cardiac rehabilitation program. During the first 6 weeks of the cardiac telerehabilitation program, the intervention group continued to receive centre-based cardiac rehabilitation but were also taught how to use the computer-based software and accelerometer. Upon initiation of the telerehabilitation only phase, they were provided with customised exercise programs and were asked to self-monitor their activities using an accelerometer. They were asked to upload their activity data to a secure webpage every two weeks. A semi-automatic tele-coaching system provided feedback on their physical activity performance, and smoking and dietary modification advice weekly via email or text message. In essence, while utilising a hybrid cardiac rehabilitation format, the intervention focussed more on sustaining behaviour change beyond attendance at a centre-based program.

### Centre-based cardiac rehabilitation in Australia

Our model’s control group aimed to reflect current Australian practice for centre-based cardiac rehabilitation. We used a recently conducted study to determine the length and number of sessions per week in Australian practice [27]. The study reported that patients had, on average, ten cardiac rehabilitation sessions. Therefore, the control group of our study were assumed to receive ten sessions of conventional centre-based cardiac rehabilitation. In the economic evaluation, we compared cost and effectiveness of the hybrid cardiac telerehabilitation program to the current centre-based cardiac rehabilitation care in Australia.

### Target population

The target population was people who are eligible, referred and compliant with attending cardiac rehabilitation in Australia. As the Telerehab III study was used as a basis for our model, we assumed that our target population had similar eligibility criteria for cardiac rehabilitation i.e. had coronary heart disease or chronic heart failure (NYHA I, II, and III). This is consistent with cardiac rehabilitation eligibility criteria reported by Australian programs [27].

Several studies, such as an audit of South Australian cardiac rehabilitation programs, have reported a high percentage of participants successfully completing telerehabilitation programs, with proportions exceeding 85% [28, 29]. However, we assumed a more conservative completion rate of 80%, for the base analysis. Furthermore, we performed a scenario analysis to assess the cost and outcome implications if 90% of participants successfully complete telerehabilitation.

### Model structure

**Fig. 1:**
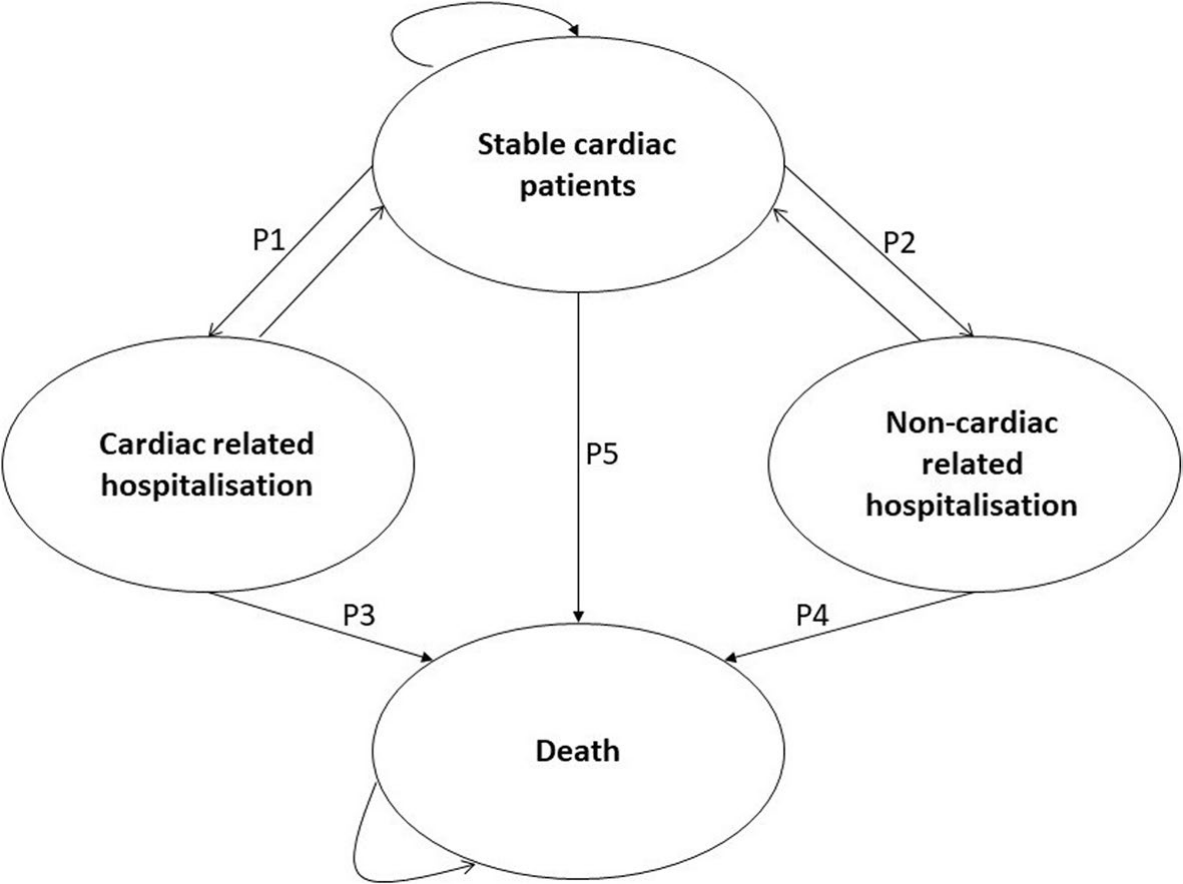
Markov model with the health states and possible transitions

A Markov model was developed on Tree Age Pro 2022 to estimate the costs and quality-adjusted life-years (QALYs) of people who had undergone hybrid cardiac telerehabilitation compared to those who underwent centre-based cardiac rehabilitation. The Markov model has four health states (Fig. 1): stable after the initial cardiac event, hospitalised due to cardiac event, hospitalised due to non-cardiac event, and death. The model starts at the "stable" health state, and the patients remain in this state until they have a cardiac-related hospitalisation, non-cardiac-related hospitalisation or die over the course of time. If they transit from “stable” to either of the hospitalised health states, they can die while being hospitalised or return to the "stable" health state.

The data used for the model was from the Telerehab III study [25] and was supplemented with inputs from other published studies [27, 30] and the Victorian Cardiac Outcomes Registry [31]. To be consistent in our study, parameter data were collected from an Australian setting wherever possible. We used a pre-COVID-19 pandemic data baseline period, as hospitalisations for non-COVID diseases decreased after 2020, and the mortality rate was higher than expected.

Effectiveness of the Telerehab III intervention was only available for two years after the intervention ended and there was a lack of data supporting longer term effectiveness. Therefore, the decision-analytic model was simulated only for a 5-year time horizon, transitioning in 1-month cycles through the health states described earlier. Quality-adjusted life-years were used to evaluate effectiveness where one QALY is equivalent to one year in perfect health. A healthcare payer perspective was taken for this analysis. An annual rate of 5% was used to discount costs and QALYs in the base case and probabilistic sensitivity analyses.

### Data sources

#### Cost information

All costs are reported in 2022 Australian dollars for the current analysis. The Campbell & Cochrane Economics Methods Group—Evidence for Policy and Practice Information—Centre (CCEMG-EPPI) Cost Converter was used when required [32]. The cost of standard centre-based cardiac rehabilitation in Australia was calculated using the National Efficient Price (NEP) Determination 2022–23-Price Weight Tables [33]. We used Appendix K, price weights for non-admitted patients—Tier 2 V7.0, to estimate the cost of cardiac rehabilitation in the model. Clinic 40.21 is Cardiac Rehabilitation and has a price weight of 0.0407. (Cost of one session is 0.0407 X NEP ($5,797) = $235.94). Therefore, assuming that the mean length of standard cardiac rehabilitation is 10 sessions [27], the cost of completing a course of centre-based cardiac rehabilitation in Australia is estimated to be about $2,359 per patient.

The cost of the resources used in the cardiac telerehabilitation program was based on the Telerehab III study [25] and included the cost of accelerometers, software, the web-page service, and information brochures (Table 1). The cost of the web-page service and information brochures was obtained from data provided in the Telerehab III study [25]. Since the accelerometer used in the Telerehab study (YorBody accelerometer) is not available in Australia, we used the Actigraph Monitor, a device with similar physical activity measurement features. The Actigraph monitor, software, and wearable accessory costs were obtained directly from the Actigraph Sales Team in March 2022.

**Table 1:** Per-cycle parameter estimates used in the model and sensitivity analysis

**Parameter**	**Baseline estimate**	**Values for sensitivity analysis**			**Source**
		**Mean**	**SEM**	**Distribution**	
**Monthly transition probabilities**					
Probability of death after a cardiac related hospitalisation	0.0032	0.0032	0.0003	Beta	[34]
Probability of death after a non-cardiac related hospitalisation	0.0006	0.0006	0.0001	Beta	[34–36]
Probability of death among stable cardiac patients	0.0018	0.0018	0.0002	Beta	[34–36]
Probability of non-cardiac hospitalisation	0.038	0.038	0.0039	Beta	[37]
Probability of cardiac hospitalisation					
Intervention	0.0089	0.0089	0.0009	Beta	[25]
Control	0.0214	0.0214	0.0022	Beta	
**Utility**					
Stable Cardiac	0.86	0.86	0.011	Beta	[30]
Hospital admission for cardiac event	0.75	0.75	0.009	Beta	[30]
Hospital admission for non-cardiac event	0.75	0.75	0.009	Beta	[30]
**Cost (in $AU 2022)**					
Cost of managing a stable cardiac patient in Australia (per month)	$122	$122	$19	Gamma Distribution	[30], MBS items 23 and 116
Cardiac related hospital admission (per admission)	$6,961	$6,961	$945	Gamma Distribution	Linked dataset
Non-cardiac related hospital admission (per admission)	$1,956	$1,956	$299	Gamma Distribution	Linked dataset
Control: completed centre-based cardiac rehabilitation program cost (per patient) *Cast of a cardiac rehab session (C) x number of sessions per week (N) x Duration in weeks (D)*	$235.94 (C) X 1.45 (N) X 7 (D) = $2,395	$235.94 X 1.45 (SD 0.5) X 7 (SD 1.11)		Gamma Distribution	[27, 33]
Intervention: completed hybrid cardiac rehabilitation program cost (per patient)	$6,255.46	$6,255.46	$957.47	Gamma Distribution	[25, 33]

To estimate the cost of managing a stable cardiac patient in Australia, we assumed that a patient with cardiovascular disease has four visits to the general practitioner and one specialist visit per year, based on expert opinion of the clinician members from the study team. The cost of a general practitioner visit was estimated using Medical Benefit Schedule (MBS) Item 23 and the cost of an annual specialist visit using MBS Item 116 [38]. A cardiac patient's average yearly medical cost was obtained from a published source [30]. The cost of hospitalisation from a cardiac-related or a non-cardiac cause was derived from a linked dataset [39]. This dataset has cost information of routinely collected and linked hospital admission, emergency presentation and death registry data of patients with cardiac disease in Queensland, Australia. The following four registries were linked in the dataset: Government Death Registration Data collection, Queensland Hospital Admitted Patient Data Collection (QHAPDC), Queensland Health Emergency Department Data Collection (QHEDDC), and National Hospital Costing Data Collection (NHCDC).

#### Utility information

The utility weights used in the model were sourced from previously published information [30]. This study utilised data from the Victorian Cardiac Outcomes Registry (VCOR), 30-day follow-up questionnaire given to patients following treatment for coronary artery disease in Victorian hospitals. The responses were used to generate 30-day utility scores, where a maximum value of 1 is indicative of being in full health, a score of 0 represents death, and less than 0 indicates a health state worse than death (rare).

#### Transition probabilities

We calculated five transition probabilities in the model. Monthly probability of:

1. Hospitalisation for a cardiac event (Fig. 1; P1)
2. Hospitalisation for a non-cardiac event (Fig. 1; P2)
3. Death after a cardiac-related hospitalisation (Fig. 1; P3)
4. Death after a non-cardiac-related hospitalisation (Fig. 1; P4)
5. Death among stable cardiac patients (Fig. 1; P5)

##### Hospitalisation probabilities

The probability of hospitalisation for a cardiac event was based on the Telerehab III study [25], which was obtained at one year follow-up. The annual probabilities of hospitalisation due to a cardiac event in the intervention and control groups were 0.1014 and 0.2285, respectively (Fig. 1; P1) [25]. In the absence of long-term evidence, we assumed that these probabilities would reduce annually by 10%.

We used the VCOR-30-day rehospitalisation data to estimate the probability of hospitalisation for a non-cardiac event (Fig. 1; P2) [37]. We assumed the rate of non-cardiac hospitalisations would remain constant over time.

##### Probability of death

In 2018–2019, there were 11.5 million hospitalisations in Australia where 10,372,469 were for people aged 20 years and over [35] and 160,787 hospitalisations were related to coronary heart disease [36]. Mortality data was sourced from the Australian Bureau of Statistics [34], where it is reported that 6,051 ischaemic heart disease (ICD-10 CM Codes I20-I25) related deaths occurred in hospitals. Therefore, we used the probability of in-hospital death related to coronary heart disease to be 0.038 (6,051/160,787) annually (monthly probability 0.0032 (Fig. 1; P3)).

We used a similar approach to estimate the probability of death during a non-coronary heart disease related hospitalisation using data from the Australian Institute of Health and Welfare and the Australian Bureau of Statistics. We deducted the age specific coronary heart disease related hospitalisations (160,787) from age-specific total hospitalisations (10,372,469) to estimate the number of non-coronary heart disease related hospitalisations. Mortality data was sourced from the Australian Bureau of Statistics [34], where it is reported that there were 78,688 non-coronary heart disease related age-specific (> 25 years) in-hospital deaths. The probability of death after a non-coronary heart disease hospitalisation, 0.0071 (Fig. 1; P4), was calculated by dividing the number of non-coronary heart disease deaths (78,688) by the total number of age-specific non-coronary heart disease hospitalisations (10,372,469).

The Australian Institute of Health and Welfare reports that in Australia an estimated 571,000 Australians aged 18 and over (2.9% of the adult population) have coronary heart disease [36]. In 2019, there were 12,193 non-hospitalised ischemic heart disease deaths reported in the Australian Bureau of Statistics data set [34]. Therefore, the annual probability of death among stable cardiac patients (Fig. 1; P5) was estimated to be 0.0214 (12,193 / 571,000).

### Model evaluation

The model was run over a 5-year period with monthly cycles. The transition probabilities dictate the proportion of patients who move between the health states during each cycle. As patients transition between health states, they accumulate costs and utilities (QALYs). The accumulated costs and utilities of the Telerehab III intervention and the current centre-based cardiac rehabilitation were compared, and an Incremental Cost-Effectiveness Ratio (ICER) was estimated using marginal QALYs and costs. The study used a cost-effectiveness threshold of AU$28,000 per QALY gained, suggested by Edney et al., which reflects the opportunity cost of introducing new health system investment into a constrained budget in Australia [40]. This threshold represents the maximum amount the Australian health system should pay for one additional unit of health benefit (QALY).

### Sensitivity analysis

Scenario analysis is critical in the economic evaluation of healthcare interventions to aid decision-makers in understanding the impact of uncertainty parameter estimates. An alternative scenario of 90% completion rate of the intervention was considered in the scenario analysis.

Probabilistic sensitivity analysis (PSA) was used to assess the uncertainty across all relevant parameters used in the model, and their effect on the cost-effectiveness results. To reflect the full range of uncertainty in the input parameters, each parameter for which there is uncertainty is assigned an appropriate probability distribution. In this study, the transition probabilities were assigned beta distributions as they are most suitable for binomial parameters. Costs parameters were assigned gamma distributions as they are able to reflect the skew that is often associated with healthcare costs. We performed Monte Carlo simulations with 10,000 iterations, sampling from the distributions described in Table 1.

To avoid the problems associated with interpreting negative ICERs, we used Incremental Net monetary benefit (iNMB) to evaluate the probabilistic sensitivity analyses.

The equation is:

$$iNMB=\left(WTP \times Change in QALY\right)-Change in costs$$

Incremental net monetary benefit (INMB) was used to summarise uncertainty in the cost-effectiveness results in this study. The expected maximum and minimum cost-saving, QALY gain, and net monetary benefit were estimated from the 10,000 iterations of the probabilistic sensitivity analyses.

## Results

Base case analysis found the telerehabilitation intervention was more effective but more costly than centre-based cardiac rehabilitation and was not cost-effective at a cost-effectiveness threshold of $28,000 per QALY (Table 2). For every 1,000 patients who undergo cardiac rehabilitation, employing the telerehabilitation intervention would cost $650,000 more, and 5.7 QALYs would be gained, over the five-year time horizon, compared to current practice.

**Table 2:** Cost-effectiveness results comparing the telerehabilitation intervention and current centre-based practice in Australia: base-case for five-year time horizon

Strategies	Cost *(2022 $ in millions)*	Incremental cost (2022 $ in millions)	Effectiveness	Incremental effectiveness	ICER $ per QALY
Current centre-based practice	20.0	0.65	4023	5.7	114,536
Telerehabilitation intervention	20.6		4028		

Results presented for 1,000 patients for 5-year time horizon

**Fig. 2:**
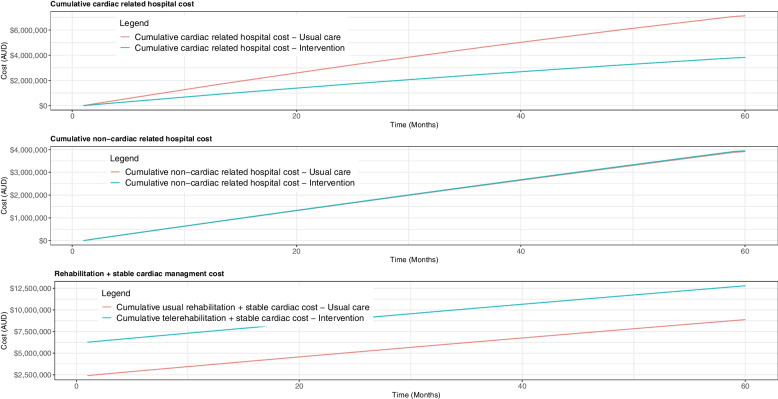
Cumulative cardiac related hospital cost, non-cardiac related cost and rehabilitation + stable cardiac management cost: Telerehabilitation intervention vs Current centre-based practice. *Results presented for 1000 patients for five-year time horizon*

Figure 2 illustrates the cumulative cardiac related hospital cost, non-cardiac related hospital cost and stable cardiac management cost that includes the rehabilitation costs as well. If a cohort of 1,000 patients undergoes cardiac rehabilitation, the intervention will reduce the cardiac-related hospital admission costs by $0.7 million alone in the first year and $3.4 million over five years. Since telerehabilitation has a substantial initial cost, the stable cardiac management cost, which includes rehabilitation expenses, remains high throughout the time horizon.

**Fig. 3:**
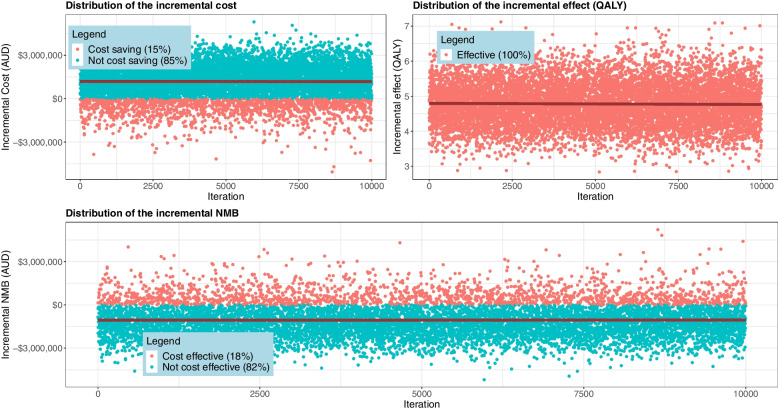
Incremental cost, QALY and net monetary benefit (iNMB) for the telerehabilitation intervention compared to the current centre-based practice. Each dot in all three graphs indicates the values generated from the 10,000 iterations in the probabilistic sensitivity analysis. Negative incremental cost indicates a cost-saving compared to current practice, and positive incremental QALY indicates more effectiveness compared to the current practice. Positive incremental NMB indicates the intervention is cost-effective compared to current practice. The solid line in the graph indicates the mean estimation

Results of the probabilistic sensitivity analysis are presented in Fig. 3. If a cohort of 1,000 patients undergoes cardiac rehabilitation with an 80% completion rate, over a five-year time horizon, implementing the new intervention would have 15% probability of being cost saving, 100% probability of being effective (QALY gain) and 18% probability of being cost-effective (positive incremental net monetary benefit) (Fig. 3). Across the 10,000 simulations, the intervention generated a maximum cost saving of $5.3 million (minimum -$5.1 million), maximum 7.1 QALY gain (minimum 2.8) and maximum $5.2 million gain (minimum -$5.2 million) in net monetary benefit over the five-year time horizon.

The results of the scenario analyses are in Table 3. This shows that even if the completion rate of the intervention is 90%, the telerehabilitation intervention is still unlikely to be cost-effective. Under PSA, the likelihood of the intervention being cost-effective, with 90% completion rate, is only 30%.

**Table 3:** Cost-effectiveness results comparing the telerehabilitation intervention and the current centre-based practice in Australia: scenario analysis for a five-year time horizon

Strategies	Cost *(2022 $ in millions)*	Incremental cost (2022 $ in millions)	Effectiveness	Incremental effectiveness	ICER $ per QALY	Probability of cost-effectiveness
Current centre-based practice	20.0	0.2	4023	6.4	38,638	30%
***Scenario analysis***	20.2		4029			
*Completion rate 90%*						

Results presented for 1,000 patients for a 5-year time horizon

## Discussion

We used a decision analytic model to assess the long-term cost-effectiveness of using a hybrid cardiac telerehabilitation program in the Australian setting. Our modelling indicates that the hybrid intervention delivered in the trial is very unlikely to be cost-effective compared to current cardiac rehabilitation practice in Australia, using a cost-effectiveness threshold of $28,000 per QALY. However, these findings should be interpreted with caution, and more research about the cost-effectiveness of context-relevant implementable hybrid models of cardiac rehabilitation is required for the reasons described below.

Various standalone cardiac telerehabilitation programs have been examined previously, with some finding the approach to be cost-effective, particularly for patients with heart failure. In the TELE-HF study in Poland where patients with heart failure underwent 8 weeks of telerehabilitation, the approach was found to be cost-effective compared to a centre-based program and the incremental cost of gaining a healthy life year ranged between US$49,832 and US$82,480 [41]. Additionally, an Australian study which investigated the cost-utility of a 12-week home-based telerehabilitation program for patients with heart failure, delivered via video conferencing, reported that the intervention had lower costs compared to a traditional centre-based program but demonstrated no significant difference in QALYs between the groups at 6-months [22]. For patients with coronary heart disease, findings about cost-effectiveness are less clear. A remotely-monitored and real-time coaching telerehabilitation approach was investigated in a New Zealand study where patients’ physical activity was monitored for 12 weeks [23]. However, no statistically significant differences in outcomes or health service utilisation were observed despite lower program and medication costs for remote cardiac rehabilitation. Unfortunately, none of the above trials provided data to model the long-term cost-effectiveness of such standalone telerehabilitation approaches.

There is evidence that a hybrid telerehabilitation approach may to be better than both stand-alone telerehabilitation or centre-based cardiac rehabilitation in preventing cardiac related hospitalisations and reducing healthcare and non-healthcare costs [42, 43]. However, it is difficult to make this argument with certainty due to the variation in the target population, type of tele-intervention, duration of monitoring and frequency of data transfer and evaluation. Additionally, the largely head-to-head comparisons of these different models seen in previous research limits understanding of how each telerehabilitation model may compare to usual care. Importantly, the two hybrid cardiac rehabilitation studies which have demonstrated benefits at one year both had extended intervention periods [25, 42]. The first was the Telerehab-III study, modelled in this paper, which comprised 12 weeks of centre-based cardiac rehabilitation (6 of which were hybrid), followed by an 18-week telerehabilitation program. This intervention was shown to reduce hospital admissions and have better health outcomes compared to 12 weeks of centre-based cardiac rehabilitation [25]. The second study, SmartCare-CAD from the Netherlands [42], used a similar approach with 6 weeks of centre-based cardiac rehabilitation and 12 weeks of virtual cardiac rehabilitation using accelerometers, data monitoring and video consultation. This was followed by 9 months of self-directed extended participation in the program at home with on-demand video coaching to maintain adherence. While there was no difference in quality of life between patients participating in centre-based and hybrid cardiac rehabilitation after one year, a hybrid model was likely to be cost-effective compared with centre-based care at a cost-effectiveness threshold of €20 000, although uncertainty remained [42].

Careful consideration of the applicability of European trialled hybrid programs to the Australian setting is also required. While there is Australian evidence to support the acceptability, adoption, and effectiveness of similar home-based coaching programs[44], this is not the case for the delivery of the centre-based component. Despite similarities in the overall core components and providers of centre-based cardiac rehabilitation in both regions, European programs generally provide a higher “dose” of intervention due to the greater number of sessions conducted[45]. For example, the centre-based component of the Telerehab III intervention (45 sessions over 12 weeks) is not indicative of the delivery of Australian centre-based cardiac rehabilitation (10–11 sessions over 7 weeks) [46, 47]. Consequently, this has implications for how we would feasibly implement this more resource intensive model into current practice, and also how the costs and effectiveness of the hybrid intervention should be interpreted in the Australian context.

These findings are important in the context of research demonstrating the relationship between the duration of cardiac rehabilitation and positive health and clinical outcomes. Longer durations of participation in centre-based or community-based cardiac rehabilitation have been shown to lead to decreased mortality, increased physical activity and sustained behaviour change after program completion [48]. Additionally, recent research has highlighted the importance of the total number of centre-based sessions attended on clinical outcomes, including a linear relationship between sessions completed and major adverse cardiac events [49]. While no research yet exists about the relationship between the frequency or duration of home-based exercise sessions and outcomes, it may be likely that it is the extended nature of these hybrid programs (rather than the delivery format) that is contributing to their comparative effectiveness. If these extended programs could however, be delivered in a cost-effective manner compared to shorter, centre-based programs, they may have an important role to play in the in the delivery of the maintenance phase of cardiac rehabilitation. Unfortunately, this does not yet appear to be the case for these models in the Australian setting. Consequently, more work needs to be done capturing cost and health utilisation data and evaluating hybrid telerehabilitation programs of shorter durations to determine if benefits remain. Most of the costs of the hybrid telerehabilitation program in our model came from the 12 weeks of centre-based sessions rather than the virtual components. If some of these in-person sessions could be reduced and replaced with virtual sessions (as in the SmartCare-CAD trial) the intervention may become cost-effective.

How program completion is defined and modelled can have substantial effects on the cost-effectiveness results. Our base case analysis assumed that the proportion of patients completing a program was 80%, yet even with a higher proportion completing (90%), the probability of the hybrid option being cost-effective was low (30%). While it would be unrealistic to assume a higher than 90% completion rate, a recent systematic review and a meta-analysis did indicate that exercise adherence is significantly higher in telerehabilitation compared to centre-based cardiac rehabilitation (standard mean difference 0.75; 95% CI 0.52 to 0.98) [13]. Several studies (including an audit of South Australian cardiac rehabilitation programs) have also reported very high percentage of participants successfully completing telerehabilitation, where the proportions have been more than 85% [28, 29].

The COVID-19 pandemic necessitated the delivery of cardiac rehabilitation by entirely virtual means for many programs, providing a potential catalyst for the adoption of such models in practice. However, uptake of telerehabilitation during this time was largely confined to phone and email modalities. A shift towards telerehabilitation interventions as described in successful trials will require significant organisational, clinical, and cultural change, particularly as issues remain around technological equity and literacy, reimbursement models, patient privacy and safety, quality of social interactions and staff capacity [16].

Additionally, recent research suggests that ongoing implementation of stand-alone cardiac telerehabilitation programs is not the preferred model of care. Rather, clinicians both in Australia and abroad have reported a desire to deliver hybrid cardiac rehabilitation programs combining in-person visits for assessments, provider consultation and initial exercise sessions with virtually delivered exercise training and education [16]. This enables a centre-based program and telerehabilitation to be run within the same site, providing patients with greater choice of modality, while allowing closer supervision of high-risk patients. The intervention we modelled in this analysis does not reflect this type of hybrid model. In fact, there is limited published evidence of any type about the effectiveness of such a model of cardiac rehabilitation, particularly the delivery of exercise and education via real-time videoconferencing [18]. Moreover, there is a paucity of research examining the ability of telerehabilitation of any type to increase the proportion of patients who enrol in cardiac rehabilitation programs.

Further research about the feasibility, effectiveness, equitability, service-level impacts and costs of telerehabilitation in Australian practice is warranted to understand the potential benefits of implementing hybrid models. Importantly, consideration of how these virtual models of care may impact underserved populations is required. While telerehabilitation may benefit those living in regional areas by improving access, it can also increase disparity for those with poor digital or health literacy, limited English proficiency, or from low socio-economic backgrounds [50]. Moreover, data is currently lacking to model the cost-effectiveness of hybrid cardiac rehabilitation programs for traditionally underserved groups such as First Nations people, culturally diverse communities or rural populations. However, if such models could increase cardiac rehabilitation program uptake amongst these groups, as well as those who decline participation in traditional centre-based programs, they may be worth implementing. Additionally, research about the preferences of patients for a hybrid telerehabilitation programme in the Australian context is lacking. These are important prerequisites to address before hybrid telerehabilitation programs have wide-spread implementation in Australia.

The current study has several limitations. Firstly, as we were not able to find trial data for an Australian cohort, the transition probabilities used in the model were derived from a range of published data sources. However, we believe that our study uses the best currently available information from published data sources representing the Australian population. Additionally, our analysis only applies to a specific application of telerehabilitation that we modelled in the study and does not imply that hybrid telerehabilitation will not be cost-effective with a different intervention or in a different context. It is hoped that two Australian telerehabilitation studies currently underway [51, 52] may soon provide additional models of hybrid telerehabilitation and/or Australian data to use in future decision analytic modelling. Secondly, a time horizon of 5 years was chosen in our model as long-term evidence of effectiveness is unavailable. The lack of long-term effectiveness data in hybrid telerehabilitation studies has a significant impact on evaluating the overall efficacy of the intervention. Thirdly, the diversity of both programs and participants across the Australian cardiac rehabilitation landscape must be acknowledged. While a standardised content outline for cardiac rehabilitation programs has been developed, there are currently no criteria for what constitutes a face-to-face program in Australia [53], and much heterogeneity exists. While we modelled an “average face-to-face program”, this is not representative of all cardiac rehabilitation services delivered and may limit generalisability of the findings across different approaches to in-person delivery. Additionally, participants attending programs have various levels of cardiac disease and risk, which is not easily captured in our utility values, effectiveness estimates and modelling. For example, we only modelled for patients with coronary artery disease because this reflects the underlying disease in the vast majority (~ 95%) of patient included in the original clinical trial. We cannot exclude that in other selected cohorts (such as those with heart failure) who may have higher risks of adverse outcomes and greater benefits from hybrid telerehabilitation that our findings may have differed.

## Conclusion

Our study is the first to evaluate long-term cost-effectiveness of a hybrid cardiac telerehabilitation intervention in the Australian context. The results indicate that over the long-term, the Telerehab III model of hybrid cardiac telerehabilitation is highly unlikely to be cost-effective compared to current centre-based cardiac rehabilitation practice in Australia. Exploration of other models of delivering cardiac telerehabilitation and their implications for the diverse population groups they are intended to serve is still required. Our findings will assist policymakers and researchers to make informed decisions when designing future trials and planning for implementation of hybrid cardiac telerehabilitation programs.

## Supplementary Information

**Additional file 1: Supplementary table 1.** The studies which were considered for the economic evaluation.

## Data Availability

This is an economic analysis based on published literature and no databases were used for analysis. The data underlying this article are available in the article. The economic analysis was performed according to the Consolidated Health Economic Evaluation Reporting Standards 2022 (CHEERS2022) Statement.
