# Supplementary material for: Hybrid cardiac telerehabilitation for coronary artery disease in Australia: a cost-effectiveness analysis
Source: BMC Health Serv Res. 2023 May 20;23:512. doi: 10.1186/s12913-023-09546-w (PMC10198753; doi:10.1186/s12913-023-09546-w)
Supplement: Supplementary file 1 — Additional file 1: Supplementary table 1. The studies which were considered for the economic evaluation. [file 12913_2023_9546_MOESM1_ESM.docx]

**Supplementary table 1:** The studies which were considered for the economic evaluation.

| **Study** | **Reason for exclusion** |
| --- | --- |
| Southard BH, Southard DR, Nuckolls J. Clinical trial of an Internet-based case management system for secondary prevention of heart disease.  Journal of Cardiopulmonary Rehabilitation and Prevention.  2003 Sep 1;23(5):341-8 | The intervention is 20 years old. |
| Cowie A, Moseley O. Home-versus hospital-based exercise training in heart failure: an economic analysis. Br J Cardiol. 2014;21(2):76. | This trial is for heart failure. A comprehensive trail-based economic evaluation has already been done in the report. |
| Maddison, R., Pfaeffli, L., Whittaker, R., Stewart, R., Kerr, A., Jiang, Y., Kira, G., Leung, W., Dalleck, L., Carter, K. and Rawstorn, J., 2015. A mobile phone intervention increases physical activity in people with cardiovascular disease: Results from the HEART randomised controlled trial. European journal of preventive cardiology, 22(6), pp.701-709 | The outcomes reported are clinical outcomes. The only ‘health service-like’ outcomes reported were “self-reported” adverse events – these are symptoms rather than health service outcomes.  Furthermore, they were not reported separately for the two groups. Therefore, this has no information for a model.  *“Twenty-two participants reported 31 serious adverse events. Of these, 15 were cardiac related (angina/chest pain = 4; stenosis = 2; heart palpitations = 1; shortness of breath = 2; dizziness = 1; pericarditis = 3; ventricular tachycardia = 2). The remaining serious adverse events included cancer diagnosis (n = 3); injury (n = 3) and other illness (n = 10). Only one serious adverse event was related to the study treatment with a participant hospitalised following a cycling accident.”* |
| Kidholm K, Rasmussen MK, Andreasen JJ, Hansen J, Nielsen G, Spindler H, et al. Cost-Utility Analysis of a Cardiac Telerehabilitation Program: The Teledialog Project. Telemed J E Health. 2016;22(7):553-63. | Limited generalisability.  No significant improvement in QALY  *“We found no significant differences in the improvement of the patients’ quality of life between the two groups (D= 0.004), and taken together, these results indicate that the intervention is not cost effective, with costs per QALY gained at more than €500,000.”*  The study follows up upon admissions, re-admissions, out-patient visits etc. However, fails to mention the causative cardiovascular reasons.  “*Only hospital visits related to heart disease are included in the study and based on the estimated* *low impact on the patients’ quality of life, no difference in these costs was to be expected.”* |
| Kraal JJ, Van den Akker-Van Marle ME, Abu-Hanna A, Stut W, Peek N, Kemps HM. Clinical and cost-effectiveness of home-based cardiac rehabilitation compared to conventional, centre-based cardiac rehabilitation: Results of the FIT@Home study. Eur J Prev Cardiol. 2017;24(12):1260-73. | Mainly clinical outcomes were presented. However, provide no information about the transition probabilities we can use for the model.  Occurrence of adverse events reported according to event characteristic, severity, and relationship to treatment. |
| Maddison R, Rawstorn JC, Stewart RAH, Benatar J, Whittaker R, Rolleston A, et al. Effects and costs of real-time cardiac telerehabilitation: randomised controlled non-inferiority trial. Heart. 2019;105(2):122-9 | The health service-related outcomes reported by Maddison et al. were only self-reported change in health state (adverse events) and HRQoL. The adverse events were reported based on severity and number of occurrences. Since the causes of hospitalisations were unclear (cannot differentiate whether cardiac-related or not), we excluded this study. |
| Cost-effectiveness of Cardiac Telerehabilitation With Relapse Prevention for the Treatment of Patients With Coronary Artery Disease in the Netherlands (Browers et al, 2021) | Participants followed up for one year, no long-term outcomes reported.  Results were statistically non-significant between the comparators. |
| Scherrenberg M, Zeymer U, Schneider S, Van der Velde AE, Wilhelm M, Van't Hof AWJ, et al. EU-CaRE study: Could exercise-based cardiac telerehabilitation also be cost-effective in elderly? International Journal of Cardiology.340:1-6. | Participants followed up for one year, no long-term outcomes reported.  Article says 15 participants in both groups were hospitalised with the causative reasons. However, an inconsistency in data reporting is noted in the control group (reported only 11). Therefore, this study was excluded.  “*The average follow-up of patients was in both groups 383 days. During the observation time, 30 patients were hospitalised for cardiovascular reasons (fifteen in both intervention group and the control group). In the intervention group, the reasons for rehospitalisation were angina pectoris with PCI (n = 4), angina pectoris without PCI (n = 7), and because of other cardiac reasons (n = 4). In the control group, the reasons for rehospitalisation were angina pectoris with PCI (n = 6), angina pectoris (n = 2), and because of decompensated heart failure (n = 3).”* |
